# Interventional Efects of the Topical of “Sanse Powder” Essential Oils Nanoemulsion on Knee Osteoarthritis in Rats by Targeting the ERS/TXNIP/NLRP3 Signaling Axis

**DOI:** 10.3389/fphar.2021.739644

**Published:** 2021-09-02

**Authors:** Zixiu Liu, Taiyang Liao, Nan Yang, Liang Ding, Xiaochen Li, Peng Wu, Peimin Wang

**Affiliations:** ^1^ Department of Orthopedics, The Affiliated Hospital of Nanjing University of Chinese Medicine, Nanjing, China; ^2^ Jiangsu Province Hospital of Chinese Medicine, Nanjing, China; ^3^ Key Laboratory for Metabolic Diseases in Chinese Medicine, First College of Clinical Medicine, Nanjing University of Chinese Medicine, Nanjing, China

**Keywords:** KOA, nanoemulsion, NEs-SP-EO, synovitis, endoplasmic reticulum stress, TXNIP/NLRP3 inflammasome

## Abstract

**Purpose:** Our recent research is dedicated to finding effective drugs for the treatment of knee osteoarthritis (KOA) from traditional Chinese medicine and trying to make full use of modern science and technology to uncover the mechanisms and targets behind them. Synovial inflammation is one of the key pathological features of KOA, and a growing number of researchers realize that early intervention of synovial inflammation may be able to reverse disease progression. The close association of traditional natural products with modern nanotechnology may be important for improving the anti-synovitis efficacy. The purpose of our research was to explore the anti-synovitis mechanism of NEs-SP-EO that might be associated with the ERS/TXNIP/NLRP3 signalling axis.

**Methods:** Chemical composition of “Sanse Powder” essential oil (SP-EO) and NEs-SP-EO were analyzed by GC-MS. NEs-SP-EO were prepared and characterized by nanoparticle tracking analysis, polydispersity index (PDI), zeta potential (ZP), ultraviolet-visible spectroscopy, and transmission electronic microscopy. The CCK8 assay for cell viability of NEs-SP-EO was performed on fibroblast-like synovial cells (FLSs) and the inflammatory environment was stimulated by LPS to explore the therapeutic mechanisms *in vitro*. Experiments of NEs-SP-EO *in vivo* were performed in male SD rats.

**Results:** The GC-MS results showed that 30 compounds were present in SP-EO and 11 components of NEs-SP-EO were identified. The results also showed that the formulation of NEs-SP-EO exhibited suitable particle size, negative charge, and stable system. *In vitro* and vivo testing, NEs-SP-EO produced anti-synovitis efficacy by reduced the induction of the ERS/TXNIP/NLRP3 signaling axis as well as regulating the overproduction of IL-1β, IL-18.

**Conclusion:** We have developed a new type of essential oil nanoemulsion from “Sanse Powder” and demonstrated that it can managing synovitis of KOA. Besides, we have initially explored the anti-inflammatory mechanism that may be related to the ERS/TXNIP/NLRP3 signaling axis.

## Introduction

KOA is currently defined as a complex, multi-system, and dynamic syndrome involving inflammation or destruction of different tissues such as synovium, cartilage, subchondral bone, and meniscus (Bannuru et al., 2019). As the number of elderly and obese people increases, the syndrome is becoming more prevalent around the world than it has been in decades, which brings a great burden to the health system and social economy (Hunter and Bierma-Zeinstra, 2019). A health care data from Sweden showed that the probability of being diagnosed with osteoarthritis exceeds 26.6% for people over 45 years old (Turkiewicz et al., 2014). Besides, osteoarthritis has become the fourth leading cause of disability all over the world in 2020, and treatment costs account for between 1 and 2.5% of GDP in high-income countries (Woolf and Pfleger, 2003; Hunter et al., 2014). However, the mechanism of KOA is complex and involves many signaling pathways. While most studies now focus on limiting synovial inflammatory responses and mitigating chondrocyte injury, our study also reveals that a series of inflammatory cascades induced by the NOD-, LRR-, and pyrin domain-containing protein 3 (NLRP3) inflammasome in FLSs ultimately amplifying KOA synovial inflammation (Zhang et al., 2019).

NLRP3 inflammasome consists of NLRP3, adaptor apoptosis-associated speck-like protein (ASC), and procaspase-1. Subsequently, procaspase-1 undergoes self-shear activation to caspase-1, which resulting in the release of inflammatory factors, leading to a cascade amplification of inflammation (Zhang et al., 2019). on the other hand, the activation of the unfolded protein response (UPR) by endoplasmic reticulum stress (ERS) is an important mechanism in the onset of KOA (Zheng et al., 2019). UPR will be activated by protein kinase RNA-like ER kinase (PERK) and inositol requiring enzyme 1α (IRE1α) (Yang et al., 2020). PERK-mediated phosphorylation of eukaryotic translation initiation factor 2α (eIF2-α) increases CCAAT/enhancer-binding protein homologous protein (CHOP), and then activation of the PERK-CHOP pathway leads to upregulation of thioredoxin-interacting protein (TXNIP) (Han et al., 2018). Elevated TXNIP protein will further activate the NLRP3 inflammasome (Zhou et al., 2010). Therefore, TXNIP serves as a “switch” and “connector” between ER stress and NLRP3 activation (Oslowski et al., 2012).

Recent research has suggested that the NLRP3 inflammasome pathway may be the mechanism by which diterpenes and other components in essential oil (EO) exert anti-inflammatory effects (Islam et al., 2020). Essential oils from natural plants offer the advantage of natural remedies over chemically synthesized drugs. Due to the chemical structure (small, volatile, and hydrophobic molecules), the EO also has a significant transdermal pro-permeation effect, penetrating cell membranes more easily and affecting various biological activities, ultimately effectively increasing the transdermal rate of topical dosage forms (de Groot and Schmidt, 2016). Besides, the great interest in EO is due to their wide range of biological activities, such as antibacterial (Rao et al., 2019), anti-inflammatory, anxiolytic effect (Zhang and Yao, 2019), analgesic (Sarmento-Neto et al., 2015), antispasmodic effect (Heghes et al., 2019), and many other effects (Labib and Aldawsari, 2015; Aziz et al., 2018). The benefits of EO have prompted people to study more efficient methods to enhance its biological activity, reduction of undesirable effects, and regulate drug release, such as the application of nanotechnology.

In recent decades, the combination of nanotechnology and natural products to develop a new drug delivery system helps to solve the problem of volatile and uneven essential oil. Specifically, nanoemulsions are transparent or translucent emulsification systems in the form of drops, which can improve the bioavailability of natural herbal products (Quagliariello et al., 2020). The encapsulation of essential oil in the nanosystem will provide better stability and avoid possible degradation and environmental factors (de Matos et al., 2019). Besides, the use of essential oil nanoemulsion as a topical drug delivery vehicle can increase drug permeability, protect the drug from volatilization and oxidation, reduce toxicity and irritation and provide more pleasant sensory properties (Dal Mas et al., 2016; Sabjan et al., 2020).

“Sanse Powder” from natural herbal products has been used for decades in Jiangsu province hospital of Chinese medicine (Mao et al., 2014). Our previous study demonstrated that “Sanse Powder” can improve synovial inflammation and enhance cold pain sensitivity and mechanical pain sensitivity through the HMGB1 pathway (Wu et al., 2019), However, it is unclear whether stable nanoemulsion can be prepared to help further investigate the anti-inflammatory mechanism of NEs-SP-EO. In the present investigation, we first clearly analyzed the components of the SP-EO and NEs-SP-EO. Next, we nano-processed the essential oil and observed *in vivo* and *in vitro* experiments that its anti-synovial efficiency may be related to reduced the induction of the ERS/TXNIP/NLRP3 signaling axis. In conclusion, “Sanse powder” Essential Oils Nanoemulsion can managing synovitis of KOA by targeting the ERS/TXNIP/NLRP3 signaling axis, which is expected to be a potential treatment in the clinic.

## Material and Methods

### Reagents

Tween-80, span-80, and octyldodecanol were supplied by Evonik Industries AG (Germany). Caprylic acid capric triglyceride (GTCC) was purchased from BASF SE (Germany). Butylated hydroxytoluene (BHT) was obtained from Sinopharm Chemical Reagent Co., Ltd. (Shanghai, China). PPG-26-Buteth-26 was obtained from GATTEFOSSE (Shanghai, China). Cell-Counting Kit-8 (CCK8) and D-Hanks were obtained from Solarbio (Beijing, China). Carbomer 940 and 4-phenyl butyric acid (4-PBA) were purchased from RHAWN (Shanghai, China). Medical breathable adhesive tape was purchased from Hongsheng Medical Technology Co., Ltd. (Shanghai, China). HE, Masson, and Sirius red staining kits were obtained from Servicebio (Wuhan, China). Antibodies for P-PERK, P-IRE1α and CHOP were purchased from Proteintech Group (Rosemont, IL, United States). Primary antibodies for TXNIP and goat anti-rabbit IgG H&L (HRP) were purchased from Affinity Biosciences (Cincinnati, OH, United States), NLRP3 and caspase-1 antibodies were obtained from SAB (Maryland, United States). CultureSure Freezing Medium was purchased from FUJIFILM Wako Pure Chemical Corperation (Guangzhou, China). Type I collagenase, LPS, and monoiodoacetic acid (MIA) were obtained from Sigma-Aldrich (Sigma, St. Louis, MO, United States). 5×HiScript II qrt SuperMix (Reverse transcription reagent) and TRIzol were obtained from Vazyme (Nanjing, China). Hieff qPCR SYBR Green Master Mix (Low Rox Plus) was obtained from Yeasen (Shanghai, China). RIPA Lysis and Extraction Buffer, DMEM, and FBS were purchased from Gibco (Rockville, United States). Besides, ELISA kits for IL-1β and IL-18 were supplied by Jinyibai Biotechnology Co., Ltd. (Nanjing, China). The primers were supplied by Sangon Biotech (Shanghai, China).

### Extract Preparation From Sanse Powder

“Sanse Powder” (batch No. 2010020) was obtained from Preparation Department, Affiliated Hospital of Nanjing University of Traditional Chinese Medicine (Jiangsu pharmaceutical system number Z04000566). The herbal medicine (Sanse Powder) is composed of twenty individual herbs (detailed information are shown in Table 1). In clinical use, the above doses of herbs are finely powdered and sterilized with cobalt 60. To obtain the essential oil extract, 150 g Sanse Powder was extracted in water distillation method using a PTHW type thermoregulated electric heating set device (Nanjing Nan’ao Technology Co., Ltd.) attached to a round bottom flask (5 L) with 3,000 ml of deionized water (5 h) at moderate temperature. The essential oil was dehydrated by adding a certain amount of anhydrous sodium sulfate and stored at 4°C away from light until it was used for nanomaterial analysis and biological assays.

**TABLE 1:** Composition and doses of “Sanse Powder.”

Chinese name	Botanical plant name	Botanic family	Amount (g)	Batch number
Man Jing Zi	*Vitex trifolia* L	Lamiaceae	40	200802
Zi Jing Pi	*Cercis chinensis* Bunge	Leguminosae	40	200801
Dang Gui	*Angelica sinensis* (Oliv.) Diels	Apiaceae	10	20061406
Mu Gua	*Carica papaya* L	Rosaceae	10	200501
Zi Dan Shen	*Salvia miltiorrhiza* Bunge	Lamiaceae	10	20201001
Chi Shao	*Paeonia veitchii* Lynch	Paeoniaceae	10	200803
Bai Zhi	*Angelica dahurica* (Hoffm.) Benth. and Hook.f. ex Franch. and Sav	Apiaceae	10	20032701
Jiang Huang	*Curcuma longa* L	Zingiberaceae	10	2009132
Du Huo	*Heracleum hemsleyanum* Diels	Apiaceae	10	200502
Gan Cao	*Glycyrrhiza uralensis* Fisch	Leguminosae	2.4	200703
Qin Jiao	*Gentiana macrophylla* Pall	Gentianaceae	4.8	19122801
Tian Hua Fen	*Trichosanthes kirilowii* Maxim	Cucurbitaceae	10	200804
Chuan Niu Xi	*Cyathula officinalis* K.C.Kuan	Amaranthaceae	10	20200401
Chuan Xiong	*Ligusticum striatum* DC.	Apiaceae	4.8	20201002
Lian Qiao	*Forsythia suspensa* (Thunb.) Vahl	Oleaceae	4.8	201001
Wei Ling Xian	*Clematis chinensis* Osbeck	Ranunculaceae	10	200502
Fang Ji	*Stephania tetrandra* S.Moore	Menispermaceae	10	200401
Fang Feng	*Saposhnikovia divaricata* (Turcz.) Schischk	Apiaceae	10	200804
Qiang Huo	*Notopterygium incisum* K.C.Ting ex H.T.Chang	Apiaceae	10	20200901
Ma Qian Zi	*Strychnos nux-vomica* L	Loganiaceae	10	190803

### Quality Control of Sanse Powder and NEs-SP-EO by Gas Chromatography-Mass Spectrometry

The chemical composition of Sanse Powder and NEs-SP-EO were analyzed on an Agilent 7890A-5975C gas chromatograph-mass spectrometer (GC-MS). We separated essential oil on an HP-5 MS column with specifications: 30 m length, 0.25 mm i. d., and 0.25 μm film (Thermo Fisher Scientific, Waltham, MA, United States). We operated the spectrometer in the electron ionization mode at 70 eV and set the scan range at (m/z) 30-500. The ion source temperature was 230°C and the inlet temperature was 250°C. We injected 1 µL of the samples at a shunt ratio of 1:20. We used the following programmed operating conditions: 45°C for 2 min, 10°C/min to 100°C for 5 min, 5°C/min to 200°C for 5 min, total running time 52 min. Helium carrier gas at 1 ml/min. We identified the resultant peaks using Agilent Mass Hunter Qualitative Analysis B.06.00 software (www.agilent.com) and each component detected in the total ion chromatogram was searched through the NIST11 database and PubChem database (>80% match). The chromatographic peaks were calculated by the area normalization method to obtain the percentage of each component.

### Formulation Technique of NEs-SP-EO

In this study, the process of formulating nanoemulsion is a two-step procedure. Briefly, firstly, the screened emulsifiers (tween-80, span-80), co-emulsifiers (octyldodecanol, PPG-26-Buteth-26), and BHT were mixed and sonicated by using a JY92-l probe ultrasonicator (Xinzhi Biotechnology Co., Ltd., Ningbo, China) at 40 kHz amplitude ultrasound for 5 min as the A phase. Then, GTCC was heated and stirred until molten and the appropriate amount of essential oil was added as the B phase. The isothermal phase A is then slowly added to phase B and stirred to form an emulsion mixture. Secondly, the emulsion mixture was homogenized three times at 120 Pa in a JN-02HC high-pressure homogenizer (Juneng Biotechnology Co., Ltd., Guangzhou, China). After cooling, NEs-SP-EO was obtained by filtration through a 0.45 μm microporous filter membrane. The NEs-SP-EO formulation ratios are shown in Table 2.

**TABLE 2:** Formulation of NEs-SP-EO.

Ingredient	Ratios (%)
GTCC	10
Tween-80	50
Span-80	5
BHT	0.5
Octyldodecanol	12.5
PPG-26-Buteth-26	2
SP-EO	20

### Characterization of Nanoemulsion

The morphology and structure of NEs-SP-EO were observed by a JEM1230 transmission electron microscope (JEOL, Tokyo, Japan), with an accelerating voltage of 120 kV. The NEs-SP-EO were diluted in ddH_2_O water (1:20 v/v) and then the mixture was dropped onto the copper mesh. After 10 min, the liquid was blotted up with filter paper and dried at room temperature. Finally, the images were taken at 30,000 times magnification. The particle size values, PDI, and ZP of NEs-SP-EO were calculated in triplicate by dynamic light scattering, using a NanoZS90 Zeta Potential Analyzer (Malvern Instrument Inc., Malvern, UK). An ultraviolet-visible absorption spectrum was obtained using a UV-2401 spectrophotometer (Shimadzu, Tokyo, Japan).

### *In vivo* NEs-SP-EO Study in a Rat Model

Topical administration of NEs-SP-EO to the knee joint was performed on the rat model following the Guidelines for Care and Use of Experimental Animals of Nanjing University of Chinese Medicine, approved project number: 013034003002. Animals were purchased from Nanjing Qinglongshan Animal Farm (License number: SCXK-SU-201-0001). 8–10 weeks male SD rats, weighing 185–205 g, were housed in an SPF-grade environment with controlled temperature and humidity and an alternating 12/12 h light/dark cycle. After 1 week of environmental adaptation, rats were randomly divided into four groups: Normal group (*n* = 10), KOA group (*n* = 10), NEs-SP-EO group (*n* = 10), and 4-PBA group (*n* = 10). The KOA model was induced by intra-articular injection of MIA (50 µL) at a concentration of 40 mg/mL. As a control, rats in the other group were injected with 50 µL of sterilized saline. Next, 4-PBA solution of 80 mg/kg was prepared (b.w. i. p. in 37°C salines) and the pH was set to 7.4 using sodium bicarbonate (Jain et al., 2016). The animals were administrated intraperitoneally with 4-PBA for 28 days after injection of MIA. Besides, studies have suggested that doses of 20–120 mg/kg/day of 4-PBA are reliable for use *in vivo* models (Qi et al., 2004; Jain et al., 2016). At the same time, The NEs-SP-EO group received topical application of NEs-SP-EO hydrogels (carbomer coated with 2 mg SP-EO) while the other three groups received carbomer hydrogel only, for 8 h each day. After 28 days, we sacrificed the animals by 3% pentobarbital sodium (b.w. i. p.90 mg/kg) to harvest the synovial tissue and plasma.

### Histological Analysis

HE staining, Sirius red staining, and Masson staining of synovial tissue were performed according to the instructions of the kit. In brief, synovial tissue underwent 4% paraformaldehyde fixation, paraffin embedding, sectioning, specific staining, cleaning, and other steps. Finally, the synovial tissue was observed by a DMI3000B microscope (Leica, Germany) system, with the use of a bright field.

### *In vitro* Cell Extraction and Culture

The method of extraction and culture of FLSs is the same as previously stated (Liao et al., 2020). In brief, first, five rat knee synovial tissue was extracted in a sterile environment and washed three times with PBS. After sufficient shearing of the synovial membrane, it was digested for 2 h by adding collagenase type I at a concentration of 0.2%. The cells were then filtered through a 70 μm cell sieve and centrifuged at 1,000 rpm for 5 min 5–10 ml of medium containing 10% FBS and 1% penicillin-streptomycin solution was used to resuspend the cell precipitate and the culture medium was refreshed in the next day and then was changed every 2 days. After about a week, the cells grew all along the wall. 3rd to 6th generation FLSs were used in this assay and cell identification was carried out as described in our previous study (Zhao et al., 2018).

The cell experiments were divided into four groups: Normal group, LPS group, NEs-SP-EO group, and 4-PBA group. The FLSs were inoculated in the 6-well plate and grew to about 80% adherent. In the following experiment, 1% serum DMEM medium should be replaced. The treatment of NEs-SP-EO and 4-PBA was summarized as follows. At first, 4-PBA (5 mM), a classical endoplasmic reticulum (ER) stress inhibitor, was added to FLSs 12 h in advance. Then, LPS (5 μg/ml) was added to FLSs for 24 h, followed by ATP (4 mmol/L) for 4 h to imitate the inflammatory environment of KOA. Finally, NEs-SP-EO (1 μg/ml) was administered for 24 h in further experiments.

### Cell Viability Assays

The CCK8 kit detected the survival of synovial cells. FLSs were incubated in 96-well plates overnight and then treated with NEs-SP-EO (1, 2, 4, 8, 10, and 20 μg/ml) for 24 h. Next, 10 µL CCK-8 solution was added for 4 h at 37°C and the absorbance was recorded at 450 nm on a microplate spectrophotometer (EnSpire).

### Western Blot

Total protein of synovial tissue and cultured cells was extracted by RIPA lysate in a container filled with ice. The protein concentration was determined by a BCA protein detection kit (Beyotime, Nanjing, China) to calculate the sample loading amount of each group. 5x SDS-PAGE buffer was added to the protein supernatant and denatured at 99°C for 10 min. SDS-page gel electrophoresis was then performed in two stages (80 v, 30 min; 120 v, 1 h). PVDF membranes were selected for transfer of protein gels with different molecular weights. 5% BSA was used to block non-specific proteins and TBST was applied to wash thrice for 10 min. PVDF membranes were incubated in primary antibody (1:1,000) overnight at 4°C and secondary antibody (1:5,000) for 2 h. Finally, bands were visualized by exposure to ECL method and ImageJ 7.04 Software was applied to evaluate the overall gray value of protein bands.

### Real-Time Polymerase Chain Reaction

First, total RNA was extracted from synovial cells and tissues using the TRIzol. In order to ensure the purity of RNA, the OD value ratio (260/280 nm) should be between 1.8—2.0. Next, 4 µL of 5 × HiScript II qRT Super Mix was added to the mixture solution and the RNA was reverse transcribed into cDNA in two steps (50°C, 15 min; 85°C, 5 s). The corresponding gene sequences were searched in the GenBank database and primers were designed by using oligo v6.6 software. The primer sequences were shown in Table 3. PCR was performed by using Hieff qPCR SYBR Green Master Mix (Low Rox Plus) according to the manufacturer’s instructions. β-actin was served as a control gene, and each gene was repeated thrice in this experiment. The 2^−ΔΔCT^ data analysis method was used to calculate relative gene expression levels.

**TABLE 3:** Primers.

Gene	Forward primer (5′-3′)	Reverse primer (5′-3′)
CHOP	TAT​GAG​GAT​CTG​CAG​GAG​G	TGA​TTC​TTC​CTC​TTC​GTT​TCC
TXNIP	TCC​GAG​TGC​AGA​AGA​TCA​G	CAC​TAA​CAT​AGA​TCA​GCA​AGG​AG
NLRP3	GAG​CTG​GAC​CTC​AGT​GAC​AAT​GC	ACC​AAT​GCG​AGA​TCC​TGA​CAA​CAC
caspase-1	ATG​GCC​GAC​AAG​GTC​CTG​AGG	GTG​ACA​TGA​TCG​CAC​AGG​TCT​CG
GAPDH	GTT​GTG​GCT​CTG​ACA​TGC​T	CCCAGGATGCCCTTTAGT

### Enzyme Linked Immuno Sorbent Assay

The expression levels of IL-1β and IL-18 in the cell culture supernatant were detected by ELISA. The gradient dilution of the standards was performed at first, followed by adding 10 µL of sample and 40 µL of sample dilution to each well at 37°C for 30 min. After washing for five times, HRP-conjugated antibody working solution, substrate working solution, and termination solution were added. OD values were determined at 450 nm, and the concentration of samples in each group was calculated according to the standard curve.

### Statistical Analysis

The experimental data were performed independently at least thrice. Graphpad 7 (San Diego, CA, United States) was applied for graphs and SPSS 19.0 software was used for statistical analysis of the data. Shapiro-Wilk test and homogeneity of variances test were used to examine the normality and homogeneity of variances of the data, respectively. Data conforming to normal distribution were expressed as mean ± standard deviation (SD), where the least significant difference (LSD) test was used for those with homogeneous variances and Dunnett’s T3 test was used for those with non-homogeneous variances. *p* < 0.05 was considered a statistically significant.

## Results

### Chemical Composition of Sanse Powder Essential Oil and NEs-SP-EO

**FIGURE 1:**
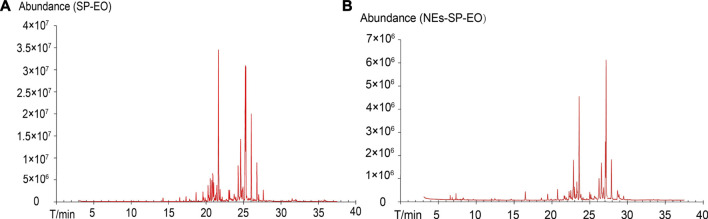
Chemical composition of SP-EO and NEs-SP-EO. **(A)** Total ion flow diagram of SP-EO. **(B)** Total ion flow diagram of NEs-SP-EO.

Firstly, we analyzed the components of SP-EO and NEs-SP-EO by GC-MS and obtained the total ion flow diagrams of SP-EO and NEs-SP-EO (Figures 1A,B). After software data processing and database query, the chemical composition tables of SP-EO and NEs-SP-EO (Table 4 and Supplementary Material) were summarized and analyzed. After identification, a total of 30 chemical components (matching degree >80%) were identified from the SP-EO, of which the top five were: ar-turmerone, (17.5739%), cadina-1 (10), (15.4345%), turmerone, (11.5095%), curlone, (7.9284%), tau-cadinol, (7.9144%). By comparing the TCMSP database (www.tcmspw.com), we found that the total content of components belonging to *Curcuma longa* L. amounted to 40.9616% (ar-turmerone, turmerone, curlone, alpha-curcumene, and beta-Turmerone). On the other hand, 11 components of NEs-SP-EO were identified, among which the total content of *Curcuma longa* L. was as high as 45.5339%. They were turmerone (27.2945%), ar-turmerone (10.5669%) and curlone (7.6721%). The main ingredients of NEs-SP-EO were consistent with SP-EO, which indicated that components belonging to *Curcuma longa* L. were detected most frequently in the SP-EO and NEs-SP-EO.

**TABLE 4:** Chemical composition of NEs-SP-EO.

Ranking	Rention time	Compound Name	Content (%)	Formula	CAS Number
1	27.1527	Tumerone	27.2945	C_15_H_22_O	180315-67-7
2	23.5874	cadina-1 (10)	19.974	C_15_H_24_	000483-76-1
3	27.0554	Ar-tumerone	10.5669	C_15_H_20_O	1000292-71-0
4	26.5368	.tau.-Muurolol	9.6175	C_15_H_26_O	019912-62-0
5	22.8365	(-)-Zingiberene	9.5974	C_15_H_24_	000495-60-3
6	27.8927	Curlone	7.6721	C_15_H_22_O	087440-60-6
7	26.2127	1,4-Cadinadiene	5.1305	C_15_H_24_	016728-99-7
8	26.8285	.alpha.-Cadinol	3.4224	C_15_H_26_O	000481-34-5
9	20.7351	Caryophyllene	2.2627	C_15_H_24_	000087-44-5
10	23.2848	Butylated Hydroxytoluene	2.2461	C_15_H_24_O	000128-37-0
11	22.9769	.alpha.-Muurolene	2.2159	C_15_H_24_	031983-22-9

### Characterization of Nanoemulsion

**FIGURE 2:**
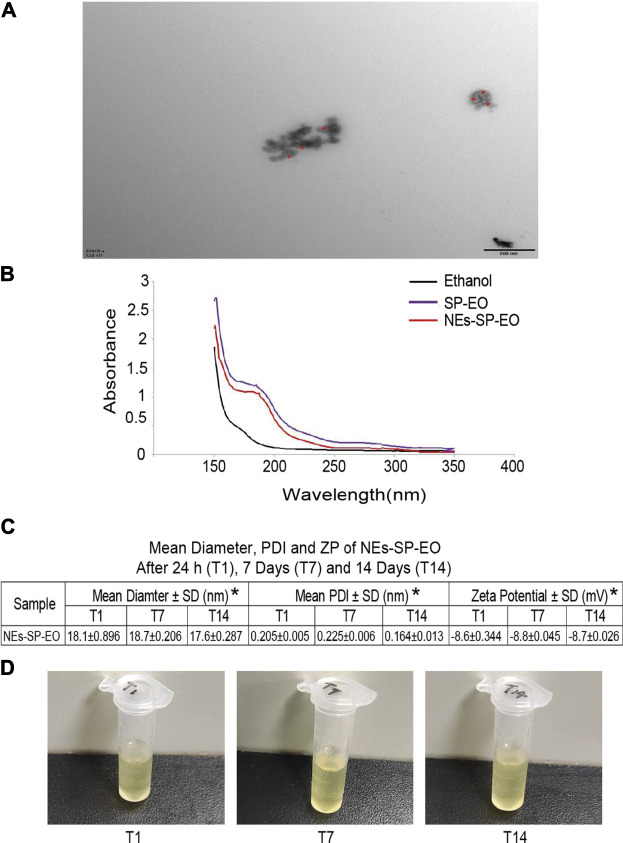
Characterization of nanoemulsion. **(A)** The morphology of NEs-SP-EO was observed by transmission electron microscopy (30,000 × 500 nm) with red arrows marking the essential oil. **(B)** The ultraviolet-visible spectroscopy of ethanol, SP-EO and NEs-SP-EO. **(C)** Mean diameter of the particle size, polydispersity index (PDI), and zeta potential (ZP) for NEs-SP-EO. **(D)** Stability observation of NEs-SP-EO on the 1st, 7th, and 14th days.

Figure 2 showed that images of the NEs-SP-EO at a magnification of ×30,000 (Figure 2A). The micrographs of the NEs-SP-EO showed a spherical circular structure. What’s more, the images of NEs-SP-EO showed possible SP-EO droplets (The red arrow indicates the location) inside the spherical circular structures. The particle size range of NEs-SP-EO was about 18.1 nm, which was confirmed by transmission electron microscopy. From the ultraviolet-visible spectroscopy, we found that SP-EO and NEs-SP-EO have similar absorption peaks (Figure 2B), and the absorption value of NEs-SP-EO was lower than SP-EO. This further confirmed the stability of the NEs-SP-EO. The formulation stability of NEs-SP-EO was assessed for 2 weeks at room temperature (Figure 2C). No signs of turbidity, foaming or phase separation were detected on the 1st, 7th, and 14th days (Figure 2D). The average particle size of NEs-SP-EO was 18.1 ± 0.896 nm on the first day, and there was no significant change in the particle size on the 7th and 14th days, which showed that the process reproducibility of NEs-SP-EO was satisfactory. The PDI of NEs-SP-EO on the 1st, 7th, and 14th days were all less than 0.3, which showed that NEs-SP-EO was distributed uniformly. Besides, the absolute values of ZP were −8.6 ± 0.344, −8.8 ± 0.045, and −8.7 ± 0.026 mv on the 1st, 7th, and 14th days, respectively, with small variations, indicating that NEs-SP-EO was stable. The results showed that the formulation exhibited suitable particle size, negative charge, and stable system.

### Anti-Synovitis Mechanisms of NEs-SP-EO *in vitro*

**FIGURE 3:**
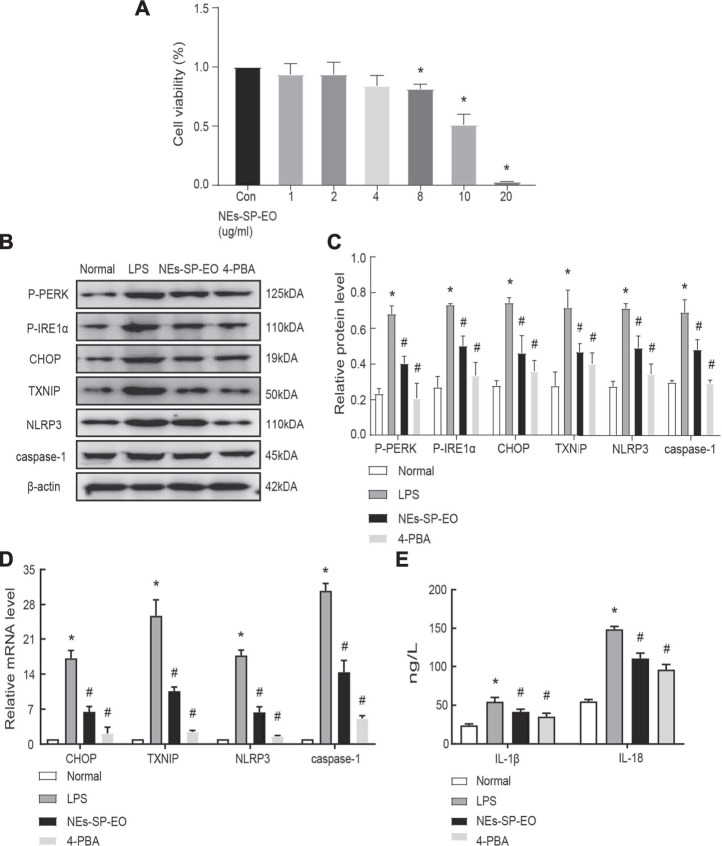
NEs-SP-EO have exerted anti-inflammatory effects of LPS-induced FLSs *in vitro*. **(A)** The CCK-8 assay showed no cytotoxicity of NEs-SP-EO at 1 μg/ml on FLSs. **(B,C)** ERS/TXNIP/NLRP3 signaling axis-related proteins and quantification analysis: P-PERK, P-IER1α, CHOP, TXNIP, NLRP3, and caspase-1 were determined by western blot. **(D)** mRNA of CHOP, TXNIP, NLRP3, and caspase-1 were suppressed by NEs-SP-EO (1 μg/ml) or 4-PBA (5 mM) treatment. **(E)** Levels of IL-1β and IL-18 in the cell supernatant were detected by ELISA. The cell experiment is expressed as the mean ± SD summarized from statistical data of three independent experiments. *p < 0.05 compared with the Normal group; #*p* < 0.05 compared with the LPS group.

Nextly, the CCK8 method was applied to explore the appropriate concentration of NEs-SP-EO, and no significant decrease in survival was observed in FLSs treated with NEs-SP-EO (0–4 μg/ml) compared to the control group, determined that 1 μg/ml was non-cytotoxic administration concentration (Figure 3A). In the following experiment, 1% serum DMEM medium should be replaced. At first, 4-PBA (5 mM), a classical endoplasmic reticulum stress inhibitor, was added to FLSs 12 h in advance. Then, LPS (5 μg/ml) was added to FLSs for 24 h, followed by ATP (4 mmol/L) for 4 h to imitate the inflammatory environment of KOA. Finally, NEs-SP-EO (1 μg/ml) was administered for 24 h in further experiments. Next, to determine the interventional effect of NEs-SP-EO on ERS/TXNIP/NLRP3 signaling axis in the LPS-induced model, the expression of relevant proteins and genes in FLSs were measured by western blot (Figures 3B,C) and qRT-PCR (Figure 3D). Compared with the normal group, proteins expression of P-PERK, P-IER1α, CHOP, TXNIP, NLRP3, and caspase-1 in the LPS group were significantly upregulated (*p* < 0.05), On the other hand, genes expression of CHOP, TXNIP, NLRP3, and caspase-1 in the LPS group were significantly upregulated (*p* < 0.05). In contrast, NEs-SP-EO and 4-PBA groups suppressed this effect. Moreover, ELISA dates demonstrated that the expression of the pro-inflammatory factors (IL-1β and IL-18) was decreased in the supernatant upon treatment with NEs-SP-EO or 4-PBA (Figure 3E).

### Preparation of NEs-SP-EO Hydrogels and Histological Analysis

**FIGURE 4:**
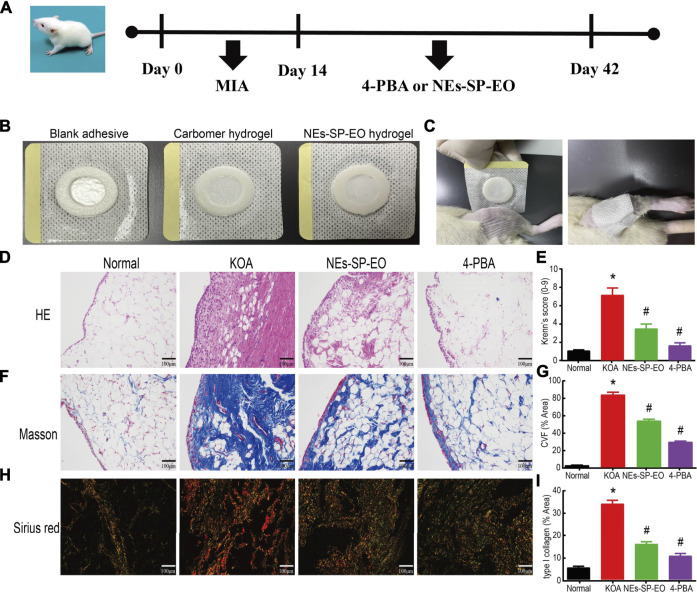
Preparation of NEs-SP-EO hydrogels and histological analysis. **(A)** Process diagram of animal experiment. **(B)** Preparation of NEs-SP-EO hydrogels. **(C)** External application of NEs-SP-EO hydrogel in rats. Histological analysis of **(D)** HE (200x, scale bar = 100 µm), **(E)** Krenn’s scores of synovitis in each group. **(F)** Masson (200x, scale bar = 100 µm) and **(G)** CVF (% Area) in each group. **(H)** Sirius red staining (200x, scale bar = 100 µm) and **(I)** type I collagen (% Area) in each group. The animal experiment is expressed as the mean ± SD summarized from statistical data of three independent experiments. *p < 0.05 compared with the Normal group; #*p* < 0.05 compared with the KOA group.

MIA (40 mg/ml) was used for modeling and 4-PBA solution (80 mg/kg) or NEs-SP-EO was applied for drug administration (Figure 4A). In order to further verify the therapeutic effect of this new formulation *in vivo*, NEs-SP-EO hydrogel (carbomer coated with 2 mg SP-EO) was applied topically to the knee joint of rats (Figure 4B). Carbomer gel changed from transparent to milky white after loading NEs-SP-EO, and the content of NEs-SP-EO in carbomer gel with SP-EO was about 2 mg. Then, it was firmly attached to the lateral side of the knee joint for 8 h every day until the end of 28 days (Figure 4C). Next, HE, Masson, and Sirius red staining methods were used to evaluate the histopathologically ameliorative effect of NEs-SP-EO or 4-PBA on synovial inflammation. In HE staining (Figure 4D), the normal group showed no thickening of cells in the synovial lining layer, neatly arranged cells, the subintimal layer was dominated by adipocytes, and almost no inflammatory cell infiltration were seen. The KOA group showed four to five layers of cells in the lining layer, disorganized arrangement, increased density and proliferation of synovial cells, and massive inflammatory cell infiltration. The rats that received NEs-SP-EO or 4-PBA exhibited less formation of lining cell layers, less resident cell hyperplasia, and inflammatory cell infiltration compared with the KOA group. Krenn’s scores were consistent with synovial pathology. (*p* < 0.05, Figure 4E). Masson staining and collagen volume fraction (CVF) were often used to evaluate synovial fibrosis (Figures 4F,G). NEs-SP-EO and 4-PBA groups had less synovial fibrosis than the KOA group (blue color indicates fibrosis), and the 4-PBA group had a better fibrosis reduction effect than NEs-SP-EO. Under polarized light microscopy, type I collagen appears as orange or bright red coarse fibers, and type III collagen appears as green fine fibers. When synovial inflammation or fibrosis occurred, the orange or bright red type I collagen fibers were abnormally increased. NEs-SP-EO and 4-PBA groups decreased the deposition of type I collagen fibers compared to the KOA group, and the 4-PBA group had a better-alleviating effect than NEs-SP-EO group (Figures 4H,I).

### Anti-Synovitis Mechanisms of NEs-SP-EO *in vivo*

**FIGURE 5:**
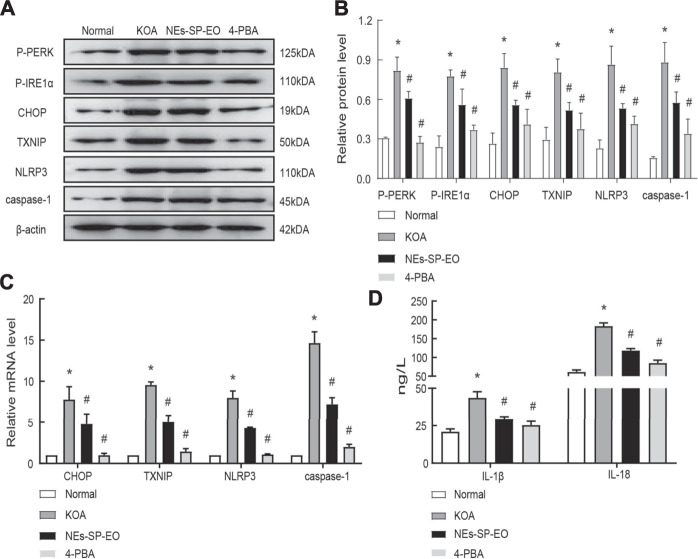
Anti-Synovitis mechanisms of NEs-SP-EO *in vivo*. **(A,B)** ERS/TXNIP/NLRP3 signaling axis-related proteins and quantification analysis: P-PERK, P-IER1α, CHOP, TXNIP, NLRP3, and caspase-1 were determined by western blot. **(C)** Genes of ERS/TXNIP/NLRP3 signaling axis were suppressed by NEs-SP-EO or 4-PBA (80 mg/kg) treatment. QRT-PCR was used to evaluate the gene changes. **(D)** IL-1β and IL-18 detection. The animal experiment is expressed as the mean ± SD summarized from statistical data of three independent experiments. *p < 0.05 compared with the Normal group; #*p* < 0.05 compared with the KOA group.

Finally, The animals were administrated intraperitoneally with 4-PBA for 28 days after knee injection of MIA. At the same time, the NEs-SP-EO group received topical application of NEs-SP-EO hydrogels (carbomer coated with SP-EO, 2 mg SP-EO) while the other three groups received carbomer hydrogel only, for 8 h each day, a total of 28 days. Next, examination of ERS and NLRP3 inflammasome related proteins and genes in MIA-induced KOA model by western blot (Figures 5A,B) and qRT-PCR (Figure 5C). The results showed that the significant inhibition of proteins expression of P-PERK, P-IER1α, CHOP, TXNIP, NLRP3, caspase-1, and mRNA expression of CHOP, TXNIP, NLRP3, caspase-1 at the doses of NEs-SP-EO (NEs-SP-EO or 4-PBA) in comparison to KOA group. Besides, we found that pro-inflammatory cytokines increased in the KOA group, while NEs-SP-EO or 4-PBA inhibited this upregulation (Figure 5D).

## Discussion

In this study, we sought to investigate the effect and anti-synovitis mechanism of NEs-SP-EO as a potential treatment *in vitro* and *in vivo*. First, the essential oil of “Sanse Powder” was extracted using the water distillation method and a new type of essential oil nanoemulsion was successfully developed from “Sanse Powder.” It is worth stating that the development of this nanosized essential oil was the result of a complex series of screenings, studies, and experiments to obtain a stable formulation, taking into account the efforts of many researchers (Trotta et al., 2003; Mu and Holm, 2018). Interestingly, for the chemical composition analysis of SP-EO and NEs-SP-EO, we found that the total contents of SP-EO and NEs-SP-EO belonging to *Curcuma longa* L. were 40.9616 and 45.5339%, respectively. *Curcuma longa* L. has become one of the most studied plant materials because of its edible and medicinal value and is widely grown in tropical and subtropical regions (Priyadarsini, 2014). What’s more, *Curcuma longa* L. is widely used in traditional medicine in Asia and Africa for inflammatory diseases, pain relief, cough, trauma, and disease prevention (Dosoky and Setzer, 2018). Modern studies have further shown that the biological activity of *Curcuma longa* L. can be attributed to volatile terpenoids (Dosoky et al., 2019; Kotra et al., 2019). Although “Sanse Powder” has been used clinically for decades, yet its target of action is still unclear. The results of SP-EO and NEs-SP-EO chemical composition analysis suggest that the effectiveness of “Sanse Powder” in the treatment of synovial inflammation may be inextricably linked to the efficacy of *Curcuma longa* L., which provides a basis and evidence for further new drug development and innovation. However, it cannot be assumed that the most abundant component of a mixture is the active ingredient. It cannot be assumed that any mixture of ingredients is more effective than either component alone. A thorough characterization of the pharmacological properties of the mixture and its components alone and in different combinations is required to determine whether there are benefits in using the mixture or individual components. This complex but highly significant work will be explored in our future research.

Next, transmission electron microscopy images of NEs-SP-EO showed the possible presence of SP-EO droplets in a spherical circular structure, which indicated that EO was encapsulated in a blank nanocarrier matrix (Gaspar et al., 2020). We next performed a series of characterization experiments on NEs-SP-EO (nanoparticle tracking analysis, PDI, ZP, and ultraviolet-visible spectroscopy). We found that SP-EO and NEs-SP-EO have similar absorption peaks. In the 14 days observation, there was no significant difference in the particle size of NEs-SP-EO, which was about 18.1 nm. The PDI of NEs-SP-EO on the 1st, 7th, and 14th days were all less than 0.3. It should be noted that PDI is an important concept in polymer science and serves as a parameter to assess the relative uniformity of particle distribution in the sample (Jantrawut et al., 2018). The negative charge of NEs-SP-EO may be related to the presence of free esters in the lipid nanosystem, which predisposes to negative charge generation (Kotynska and Figaszewski, 2018). No significant variations were discovered between time 0–14 days, in relation to the mean particle diameter, PDI, and ZP, which demonstrates the stability of NEs-SP-EO.

Further, we performed anti-inflammatory experiments in rat synovial cells. CCK8 results showed that 0–4 μg/ml of NEs-SP-EO did not produce significant cytotoxic effects on FLSs. In particular, FLSs are crucial effector cells in the progression of synovitis (Mathiessen and Conaghan, 2017). Next, we demonstrated at the cellular level that the anti-inflammatory mechanism of NEs-SP-EO may be related to reduced the induction of the ERS/TXNIP/NLRP3 signaling axis. Many studies have surfaced on the possible association of essential oil anti-inflammation with the NLRP3 inflammasome pathway (Islam et al., 2020; Yin et al., 2020). Some papers reported that TXNIP is a critical signaling node that links ER stress and inflammation. TXNIP is induced by ER stress through the PERK and IRE1 pathways, induces IL-1β mRNA transcription, activates IL-1β production by the NLRP3 inflammasome (Lebeaupin et al., 2015; Samidurai et al., 2021). In the field of osteoarthritis, TXNIP/NLRP3 signaling pathway is closely related to chondrocytes and synoviocytes, but further investigation is needed (Li et al., 2016; Gu et al., 2019). In this study, we verified in rat synovial tissue and FLSs that inhibition of endoplasmic reticulum stress (4-PBA) would reduce TXNIP and NLRP3 expression. The possible mechanism of TXNIP activation of the NLRP3 inflammasome is that TXNIP release from the nucleus will interact with the leucine-rich repeat (LRR) domain at the C-terminus of the NLRP3 sensor (Zhou et al., 2010). It is worth emphasizing that the role of TXNIP in NLRP3 response is also controversial (Muri et al., 2020). In short, this experiment preliminarily observed the intervention effect of NEs-SP-EO on the above linkage pathway, which was further verified in animal experiments.

Topical administration of drugs includes dermal and mucosal administration (e.g., skin, nose, eyes, rectum, etc.), among those, the transdermal route being the most widely used as the largest external receptor (Leppert et al., 2018). The essential oil is transcutaneously administered through the skin to reach the target, chinese medicine and its extracts (e.g., extracted essential oil) have been used for more than 3,000 years and are often perceived to act locally in this way, but over the years it has become clear that transdermal administration can have the similar systemic effect as oral administration (Chen et al., 2019). The average particle size of nanoemulsion is only 18.1 nm, which can improve the transdermal efficiency and anti-inflammatory effect of the SP-EO, and can easily cross the skin stratum corneum without the adverse reaction and first-pass effect of nonsteroidal anti-inflammatory drugs in general.

However, there are many undesirable limitations of this study. First, pharmacodynamic and pharmacokinetic investigations of SP-EO and NEs-SP-EO seem to be insufficient, and the in-depth study in macromolecular materials and pharmacology are worth the effort, which may be limited by the lack of cross-disciplinary knowledge. Besides, the purpose of this study was to prove the superiority of the nanoemulsion, it should have been compared with the non-emulsified EO. What’s more, due to the limitation of experimental conditions, we failed to further study the characterization of NEs-SP-EO in depth. Finally, the hydrogels used in the animal experiments were prepared rudimentary and still need to be optimized for the conditions of SP-EO content. Future research will focus on solving the above limitations and try to develop patches of NEs-SP-EO for clinical applications.

## Conclusion

In conclusion, we have developed a new type of essential oil nanoemulsion from Sanse Powder and demonstrated that it can reduce the synovial inflammation of KOA. Besides, we have initially explored the anti-inflammatory mechanism that may be related to reduced the induction of the ERS/TXNIP/NLRP3 signaling axis *in vivo* and *in vitro*. Our researches may have the potential to become a clinically effective topical drug in the future.

## Data Availability

The original contributions presented in the study are included in the article/Supplementary Material; further inquiries can be directed to the corresponding author.
